# Effect of frozen storage of bovine colostrum for up to 1 year on concentrations of immunoglobulins and insulin as well as bacterial counts

**DOI:** 10.3168/jdsc.2024-0731

**Published:** 2025-03-03

**Authors:** Trent A. Westhoff, Sabine Mann

**Affiliations:** Department of Population Medicine and Diagnostic Sciences, College of Veterinary Medicine, Cornell University, Ithaca, NY 14853

## Abstract

Freezing colostrum is a common management strategy to ensure availability of high-quality colostrum for each calf. Limited data are available on the effects of freezing colostrum on important colostral components. The objective of this study was to investigate the effects of freezing bovine colostrum at −20°C for 1 yr on concentrations of IgG, IgA, IgM, and insulin, as well as on total plate count (TPC) and coliform counts (TCC). Composite colostrum samples (250 mL) were collected from the complete first milking from Holstein cows (n = 10), immediately cooled on ice, and divided into fourteen 8-mL aliquots. One aliquot was stored on ice for analysis within 6 h of collection (fresh) and 13 additional aliquots were frozen and stored at −20°C for subsequent analyses upon thawing at 21°C to 24°C in 4-wk intervals. All samples were analyzed for Brix % via a digital Brix refractometer; IgG, IgA, and IgM via radial immunodiffusion; insulin via radio immunoassay; and TPC and TCC via commercial ready-to-use aerobic bacterial culture plates. Data were analyzed using mixed effects repeated-measures ANOVA. Compared with fresh colostrum, Brix % was 4.1% ± 0.9% to 6.2% ± 0.9% lower between 4 and 52 wk. Concentration of IgG was reduced 8.1% ± 2.8%, 7.6% ± 2.5%, and 8.2% ± 2.3% at 32, 44, and 48 wk relative to sampling, respectively. Freezing colostrum did not affect concentrations of IgA or IgM. Insulin concentrations were reduced 14.5% ± 3.5% at 16 wk, 11.1% ± 3.0% at 40 wk, 18.4% ± 3.3% at 48 wk, and 20.8% ± 3.2% at 52 wk relative to sampling compared with insulin concentration determined in fresh colostrum. Coliform count was reduced 22.1% ± 7.6% by 4 wk and continued to be lower at each time point through 52 wk. Total plate count was reduced at 4, 20, and 24 wk to 46.9% (30.9%–71.4%), 47.9% (31.5%–72.9%), and 51.0% (33.3%–78.1%) of the TPC in the fresh colostrum, but did not differ at the other time points. We conclude that freezing colostrum appeared to affect Brix %, TCC, and concentrations of IgG and insulin. As such, producers should store colostrum at −20°C for no longer than 32 wk to minimize the risk of a change in the composition.

## Highlights

• Colostrum samples were stored at −20°C for 1 year.
• Frozen storage of colostrum decreased IgG, insulin, and coliform count.
• TPC, IgA, and IgM did not differ after 1 year.
• Producers should store colostrum at −20°C for no longer than 32 weeks.

## Graphical Abstract

**Figure:**
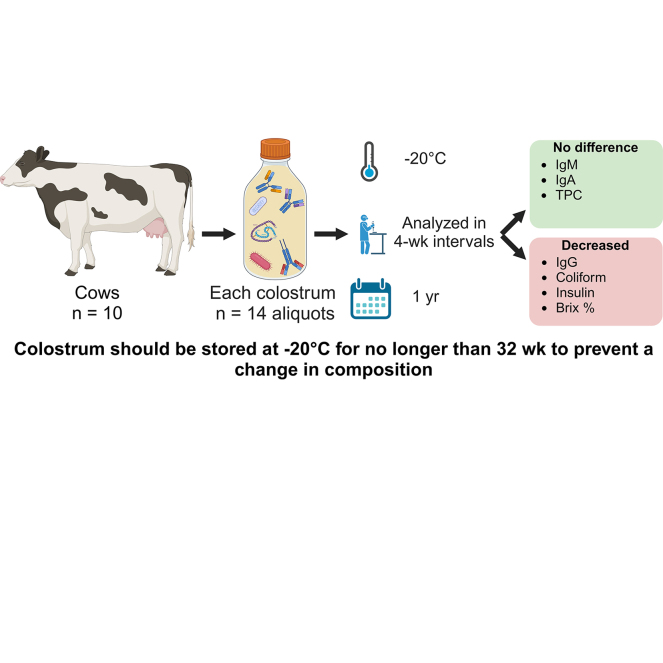
**Summary:** Colostrum samples (n = 10) were collected from Holstein cows. One aliquot remained fresh, and the remaining 13 aliquots were frozen and stored at −20°C for analysis of concentrations of immunoglobulin (Ig) G, IgA, IgM, and insulin as well as total plate and coliform count in 4-week intervals over the course of 1 year. Freezing colostrum decreased concentrations of IgG by week 32, and insulin by week 40, as well as Brix % and coliform count in all frozen samples compared with fresh samples. Storage time did not consistently influence concentrations of IgA and IgM or total plate count (TPC). The figure was created with BioRender.com.

Ingestion of an adequate volume of high-quality colostrum is fundamental to raising healthy and productive calves. Bovine colostrum contains elevated concentrations of nutrients, Ig, hormones, and growth factors (Blum and Hammon, 2000; Godden et al., 2019). Although IgG concentration has historically defined colostrum quality for its importance to transfer passive immunity, colostrum also contains IgA and IgM, albeit at lower concentrations. Their role lies in pathogen mitigation on mucosal surfaces and in the bloodstream, respectively (Bush and Staley, 1980; Lopez and Heinrichs, 2022). Moreover, interest in the concentration of non-nutritional bioactive factors, such as insulin, has grown because of its influence on intestinal maturation mediated through localized binding and stimulation of insulin receptors (Blum and Hammon, 2000; Hare et al., 2023).

On-farm storage of colostrum is a common management practice with a reported 22% to 84% of dairy producers in the United States (Kehoe et al., 2007; Westhoff et al., 2023) and Europe (Staněk et al., 2014; Ahmann et al., 2024) utilizing refrigerators and freezers for colostrum storage. Refrigeration has been recommended for short-term storage (≤2 d) only (Stewart et al., 2005) because the extent of bacterial contamination in individual colostrum pools is not commonly known on-farm and continued bacterial growth occurs. Alternatively, freezing colostrum at −20°C for up to 1 yr has been recommended for long-term storage (Schipper et al., 1981; Godden et al., 2019), but data on the effect of extended cold storage on bovine colostral components remains scarce. When storing bovine colostrum at −20°C, concentrations of IgG, IgM, IGF-1, and lactoferrin were decreased by 3 mo compared with the concentrations determined in fresh samples (Abd El-Fattah et al., 2014). Furthermore, the concentration of IgA in human colostrum was reduced by 12 mo when stored at −20°C compared with the concentration determined after freezing for 1 wk (Ramírez-Santana et al., 2012), but insulin concentration in human milk was stable for 30 mo when stored at −20°C (Mank et al., 2021). Because of the importance of colostral components such as Ig to transfer passive immunity and insulin for maturation of the intestine, as well as the detrimental effects of high colostral bacterial contamination on IgG absorption (James et al., 1981), the effects of frozen storage on colostrum components warrant further evaluation. As such, the objective of this study was to measure the effects of freezing bovine colostrum at −20°C for 1 yr on concentrations of IgG, IgA, IgM, and insulin, as well as on total plate count (**TPC**) and total coliform count (**TCC**).

This study was conducted from November 2023 to November 2024. Composite colostrum samples (250 mL; n = 10) were obtained from Holstein cows at a commercial dairy farm in New York State. First-milking colostrum was harvested from cows selected by convenience at the 0930 h milking into polypropylene milking buckets (DeLaval) on a 100-stall rotary parlor (DeLaval). Milking buckets were thoroughly washed with detergent (Expedite Liquid, DeLaval), sanitized with a diluted sodium hypochlorite solution, rinsed, and allowed to air dry after each use. Samples were immediately placed on ice for transportation (30 min) to a laboratory at Cornell University.

Upon arrival at the laboratory, colostrum was carefully mixed and divided into fourteen 8-mL aliquots. One aliquot remained on ice (fresh) and the remaining 13 aliquots were frozen and stored at −20°C for subsequent analyses. Colostrum Brix %, TPC, TCC, and concentrations of IgG, IgM, IgA, and insulin were determined in fresh colostrum within 6 h of collection as well as in 4-wk intervals over the course of a year in colostrum stored at −20°C. Frozen colostrum samples were thawed at 21°C to 24°C before analysis. Brix % was determined using a digital refractometer (model PA201, Misco) that was zero-set with distilled water before use at each time point and calibrated according to the manufacturer's instructions. Concentrations of IgG, IgA, and IgM were assessed by radial immunodiffusion (Triple J Farms) as was previously described for IgG analysis in Westhoff et al. (2024a). In brief, colostrum was warmed to 21°C to 24°C, thoroughly vortexed, and diluted with sterile saline warmed to 37°C. Five microliters of each sample and pooled reference sera were added to radial immunodiffusion plates. The precipitin ring diameter was measured in 0.1 mm increments following a 24-h incubation at 21°C to 24°C using a 10× magnified loupe equipped with an LED light and embedded scale (Ideal-tek S.A.). Fresh samples were diluted 8-fold for IgG analysis and the initial IgM and IgA concentrations were determined in samples run undiluted and diluted 2- and 4-fold. At subsequent time points, samples were diluted 8-fold for IgG analysis and either undiluted or diluted 2-, 3-, or 4-fold for IgM and IgA analysis, based on the dilution needed for each sample to fall within the linear range of detection of each assay.

For determination of insulin concentrations, the fat layer was removed from colostrum by centrifugation at 2,400 × *g* for 20 min at 21°C. Skim colostrum was diluted 40-fold with assay buffer (0.05 *M* phosphosaline, pH 7.4, containing 0.025 *M* EDTA, 0.08% sodium azide, and 1% RIA-grade BSA), and insulin concentration was determined using a rat insulin RIA kit (RI-13K; EMD Millipore Corporation) at the Endocrinology Laboratory at the New York State Animal Health Diagnostic Center (Ithaca, NY). The standard operating procedure for detection of bovine insulin followed the accredited diagnostic laboratory practices for this analyte and was performed according to the manufacturer's instructions with the exception that human insulin standards (HI-14K, EMD Millipore Corporation) were used in replacement of the kit's rat insulin standards to create a standard curve range of 3.125 to 100 µIU/mL.

Association of Official Analytical Collaboration International–validated (no. 010404 and 110402) ready-to-use aerobic bacterial plates (Compact Dry TC and Compact Dry EC, Hardy Diagnostics) were used for bacterial quantification. Plates for TPC contained dehydrated culture media, a gelling agent, and 2,3,5-triphenyl tetrazolium chloride and were previously validated against the standard pour plate method in food samples (Kodaka et al., 2005). Plates for TCC contained dehydrated culture medium, a gelling agent, 5-bromo-6-chloro-3-indoxyl-β-d-galactopyranoside, 5-bromo-4-chloro-3-indoxyl-β-d-glucuronic acid, and cyclohexylammonium salt and were previously validated against the most probable number method in food samples (Kodaka et al., 2006). Plates were inoculated with 1 mL of undiluted or diluted colostrum, incubated at 35°C, and TCC and TPC were quantified at 24 and 48 h, respectively, according to the manufacturer's instructions. The initial bacterial counts were determined by diluting fresh samples with sterile water at 50-, 150-, and 1,500-fold for TPC and 5-, 50-, and 150-fold for TCC. At subsequent time points, dilution factors were chosen to target a countable range (<250 cfu/mL). Samples were diluted with sterile water at either 50-, 75-, 100-, 150-, or 300-fold for TPC and undiluted, or at 3-, 6-, or 8-fold for coliform quantification. Sterile water used for dilutions was included as a negative control.

The sample size was determined based on the expected difference in IgG concentration in stored samples compared with fresh colostrum. We estimated that time would explain 5% of the variance and the correlation among repeated measurement was 0.8. Accounting for repeated measurements, a significance level of 0.05, and a power of 95% resulted in a sample size of 9 colostrum samples (G*Power v 3.1.9.7, Heinrich-Heine-Universität Düsseldorf). We further increased this number by 10% to reach a conservative sample size of 10 samples.

The effect of storage length on colostral components was analyzed using repeated measures ANOVA in PROC MIXED (v. 9.4, SAS Institute Inc.) with the fixed effect of time and repeated effect of time with a subject of sample ID. The model assumptions of normality and homoscedasticity of the residuals were visually assessed. Outliers were tested for using the INFLUENCE statement and datapoints with a Cook's distance >0.5 were removed. To determine the stability of each constituent, each time point was compared with fresh colostrum using Dunnett's test (Tallarida and Murray, 1986) to adjust for multiple comparisons. Data at each time point are presented as the LSM and 95% CI percentage of the concentration or count determined in fresh colostrum. Significance was declared at *P* < 0.05.

Fresh colostrum had mean ± SD (range) concentrations of IgG (118.8 ± 38.6 [48.4 to 186.1] g/L), IgM (3.9 ± 1.9 [1.8 to 7.5] g/L), IgA (6.8 ± 4.6 [0 to 14.5] g/L), insulin (1,617 ± 897 [797 to 3,530] µIU/mL), and Brix (27.4% ± 2.2% [22.8% to 30%]). Total plate count and TCC were (20,475 ± 19,657 [2,850 to 55,650] cfu/mL) and (300 ± 361 [0 to 1,000] cfu/mL), respectively, in fresh colostrum samples. Samples without detectable IgA (n = 1) and coliforms (n = 4) were removed from statistical analysis.

Freezing colostrum reduced IgG at 32, 44, and 48 wk (*P* ≤ 0.04) and Brix from 4 to 52 wk (*P* < 0.01; Figure 1) compared with fresh colostrum but did not affect IgM and IgA concentrations (*P* ≥ 0.06). Compared with fresh colostrum, TPC was lower at 4, 20, and 24 wk relative to sampling (*P* ≤ 0.01) and TCC was reduced from 4 to 52 wk relative to sampling (*P* < 0.05; Figure 2). Insulin was lower at 16, 40, 48, and 52 wk relative to sampling compared with fresh colostrum (*P* < 0.01; Figure 3).

**Figure 1:**
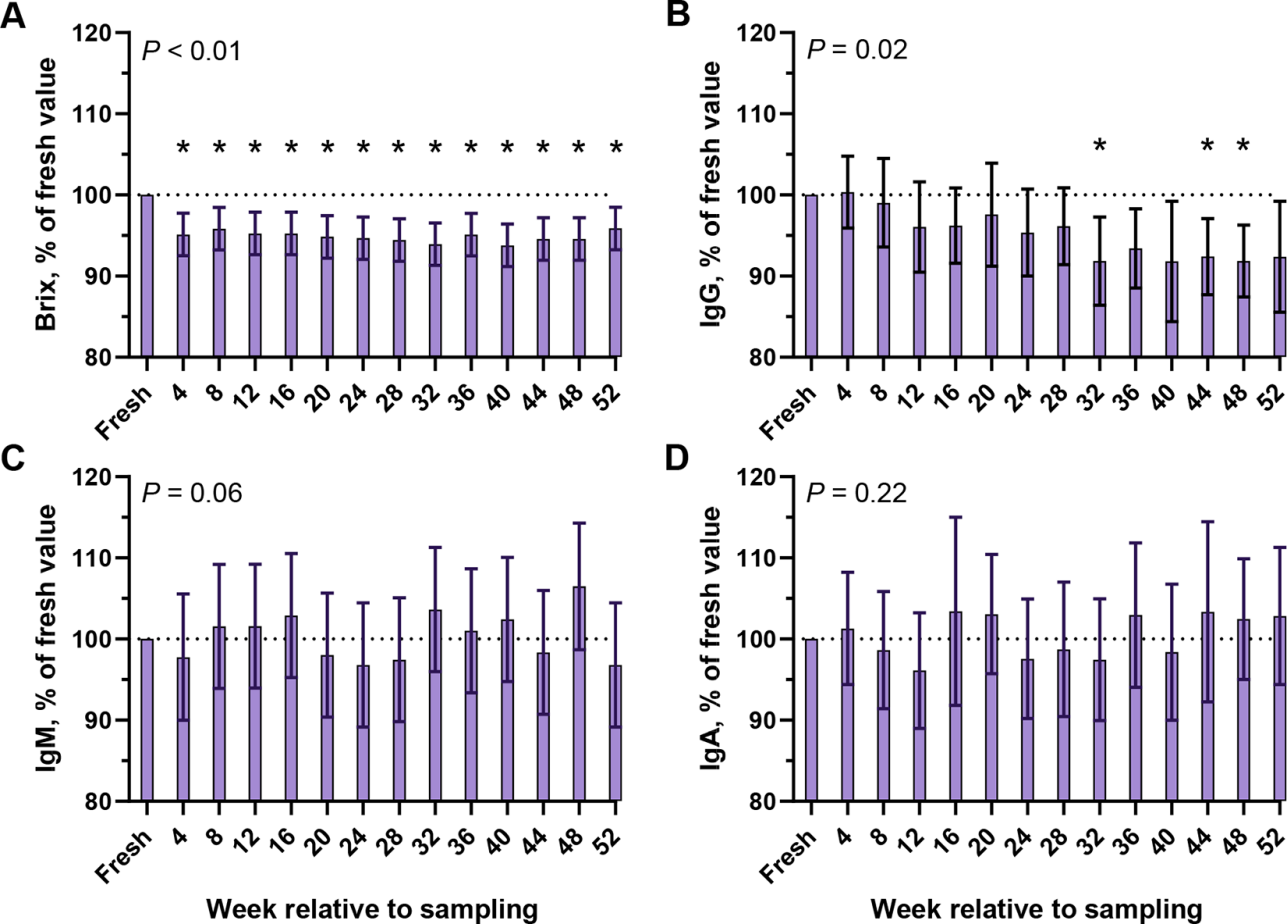
Effect of freezing bovine colostrum samples (n = 10) collected on a commercial dairy farm at −20°C for 1 yr on Brix % (A) and concentrations of IgG (B), IgM (C), and IgA (D). *P*-value denotes the effect of time. *Denotes a difference between the individual time point compared with fresh colostrum (*P* < 0.05; Dunnett's test). Data presented as LSM and 95% CI.

**Figure 2:**
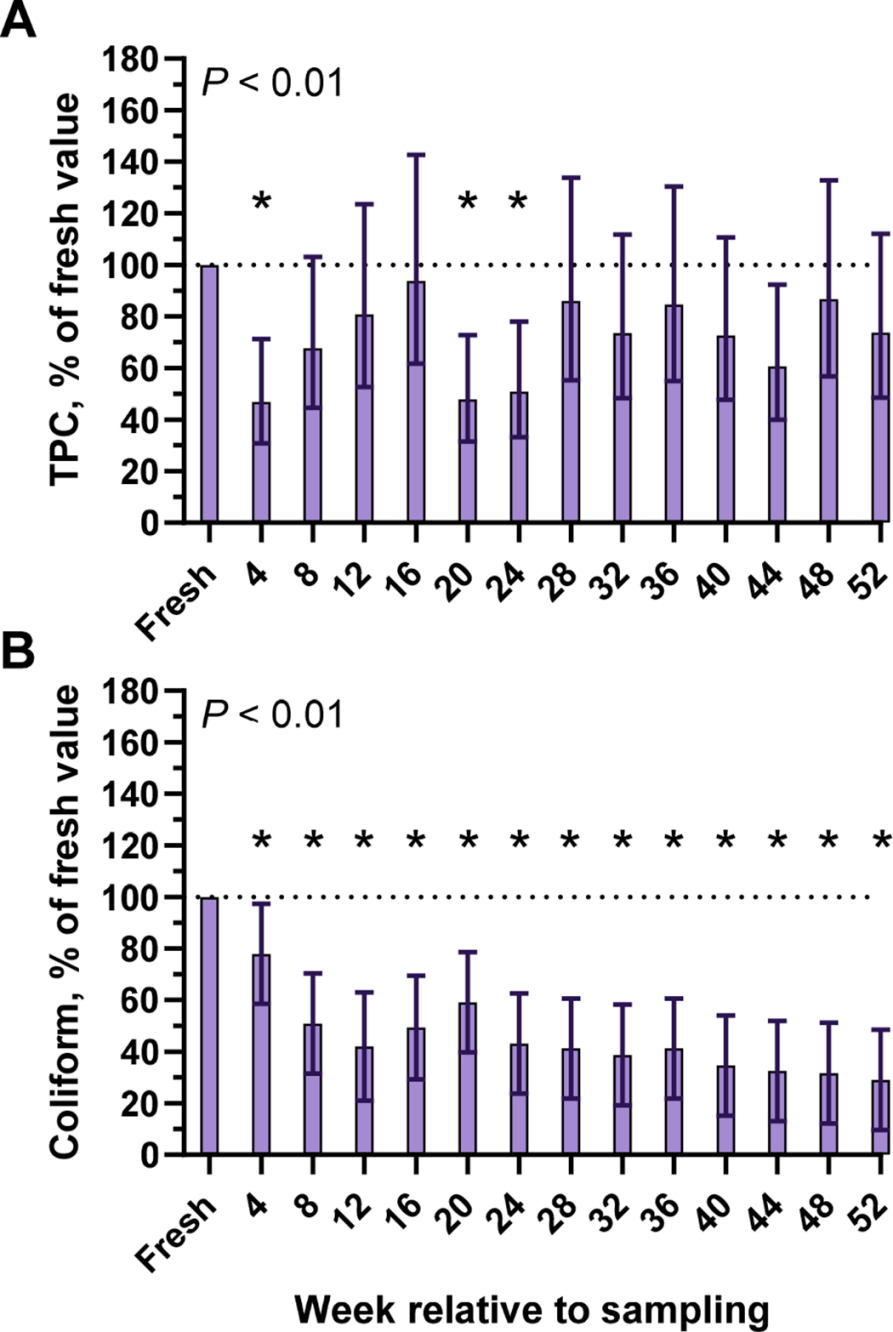
Effect of freezing bovine colostrum samples (n = 10) collected on a commercial dairy farm at −20°C for 1 yr on total plate count (TPC; A) and coliform count (B). *P*-value denotes the effect of time. *Denotes a difference between the individual time point compared with fresh colostrum (*P* < 0.05; Dunnett's test). Data presented as LSM and 95% CI.

**Figure 3:**
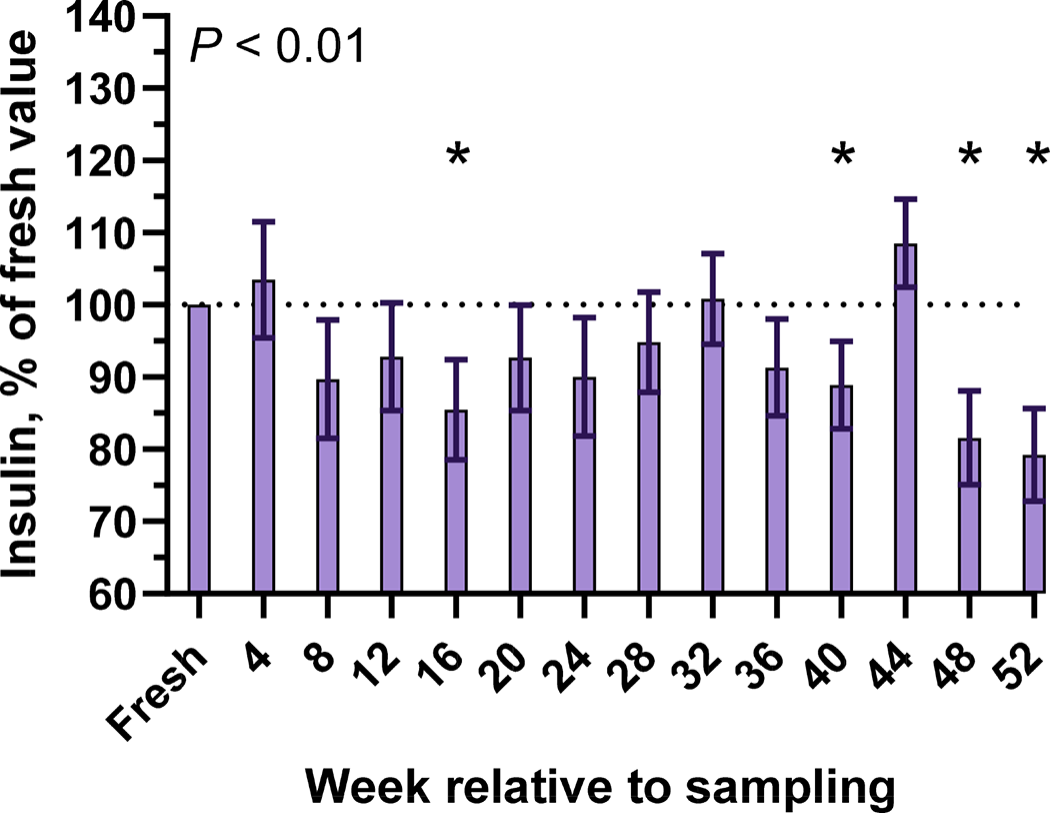
Effect of freezing bovine colostrum samples (n = 10) collected on a commercial dairy farm at −20°C for 1 yr on insulin concentration. *P*-value denotes the effect of time. *Denotes a difference between the individual time point compared with fresh colostrum (*P* < 0.05; Dunnett's test). Data presented as LSM and 95% CI.

Freezing colostrum as a long-term storage method has been adopted by producers in many regions (Westhoff et al., 2023; Ahmann et al., 2024) and is an effective practice to maintain an adequate supply of high-quality colostrum during periods of low colostrum yields (reviewed by Westhoff et al., 2024b). This study was designed to determine the stability of Ig and insulin concentrations as well as investigate changes in total bacteria and coliform counts in bovine colostrum frozen at −20°C for 1 yr.

In Abd El-Fattah et al. (2014), freezing 36 bovine colostrum samples at −20°C for 3 mo decreased IgG and IgM concentrations by 2.0 g/L (6.8%) and 2.8 g/L (39.3%) compared with samples analyzed before freezing. To our knowledge, the impact of long-term freezing on IgA in bovine colostrum has not been described. In human colostrum, IgA concentrations remained stable in samples frozen at −20°C for 1 wk and up to 6 mo, but decreased 41% at 12 mo (Ramírez-Santana et al., 2012). The extrapolation of these findings toward bovine colostrum should be done with caution. In contrast to the aforementioned studies, we did not find evidence that storing bovine colostrum at −20°C for 1 yr affected IgM and IgA, but IgG concentration was 8.1% ± 2.8%, 7.6% ± 2.5%, and 8.2% ± 2.3% lower at 32, 44, and 48 wk, respectively, compared with the concentration determined in fresh colostrum. Although an ∼8% reduction in IgG concentration in samples with an elevated initial IgG concentration (e.g., ≥100 g/L) might have lower biological implications for the calf, it remains unknown whether the initial IgG concentration influences the dynamics of the rate of decline. As such, our findings suggest on-farm protocols that store colostrum frozen for ≤32 wk would minimize the risk of reductions in IgG concentration.

Bovine colostrum contains a greater concentration of insulin (21.2 [14.8–30.3] µg/L) compared with mature whole milk (0.5 [0.3–0.7] µg/L; Fischer-Tlustos et al., 2024) and through binding and stimulation of insulin receptors has demonstrated positive effects on gastrointestinal development of neonates (Hare et al., 2023). In Mank et al. (2021), freezing human milk at −20°C for 2.5 yr did not affect the insulin concentration, but the effect of storing bovine colostrum at −20°C on colostral insulin concentrations has not been well described to the knowledge of the authors. In the current study, measured insulin concentrations were reduced 14.5% ± 3.5% at 16 wk, 11.1% ± 3.0% at 40 wk, 18.4% ± 3.3% at 48 wk, and 20.8% ± 3.2% at 52 wk relative to sampling. Although a standardized protocol was used for the duration of the study, we believe that interassay variability may have been present, specifically at 16 wk. Without measurements beyond 52 wk, it is unknown whether the measured reductions in insulin concentration at 40, 48, and 52 wk were the result of true reductions in insulin, interassay variability, or a combination of both.

Colostrum can be exposed to considerable environmental contamination from equipment used during colostrum harvest (Stewart et al., 2005; Hyde et al., 2020) that can be exacerbated when colostrum is not quickly cooled following harvest (Cummins et al., 2016). In our study, we used thoroughly cleaned and sanitized equipment to harvest colostrum and immediately cooled colostrum on ice after harvest. All samples were well below the current industry standard for TPC (<100,000 cfu/mL) and TCC (<10,000 cfu/mL; McGuirk and Collins, 2004). In Chandler et al. (2023), freezing 14 bovine colostrum samples for ≤12 h was not shown to affect TPC. However, freezing raw bovine milk decreased TPC by 4 wk (Alrabadi, 2015) and TCC by 72 h (Hubáčková and Ryšánek, 2007). It is noteworthy that the tolerance to freezing appears to be dependent on the bacterial species in colostrum (Villanueva et al., 1991; Hubáčková and Ryšánek, 2007), a phenomenon we previously also described for heat treatment of colostrum (Mann et al., 2020). Though individual species were not quantified and the samples included herein had low initial TCC, coliforms were consistently reduced at all time points between 4 and 52 wk compared with fresh colostrum, suggesting that freezing colostrum was effective at reducing the coliform load in these samples. Although TPC was lower at 4, 20, and 24 wk relative to sampling, results were highly variable between time points, and TPC did not differ from fresh colostrum from 28 to 52 wk. Thus, our data suggest that frozen storage decreased colostral TCC but did not affect TPC over 1 yr.

A limitation of the current study was that colostrum was frozen in a smaller volume (8 mL) than would typically be stored on commercial dairy farms (3–4 L). The difference in volume would affect the rate of freezing and thawing that might affect the measured constituents. The initial total bacteria and coliform counts in samples included in this study were well below the current but likely outdated industry standard maximum recommended counts and below the mean (range) TPC reported previously (79,433 [1,000–6,309,573] cfu/mL; Morrill et al., 2012). However, direct comparison with the aforementioned nationwide survey should be made with caution because the authors used different methods to select, store, transport, and analyze samples for bacterial contamination. Further, we excluded samples (n = 4) from analysis due to no detectable coliforms, which decreased our sample size for this objective and should be acknowledged when interpreting these data. Finally, although protocols were in place to reduce interassay variability between time points, insulin samples were run by different personnel and multiple assay lots in a diagnostic laboratory that might have introduced higher interassay variability than the assays performed by a single investigator and assay kits of the same lot in our own laboratory. Although we reduced interassay variability where possible in this longitudinal study, precision and accuracy of the estimates provided in this work could further have been improved if we had run each of the 10 different colostrum samples in duplicate for all outcomes of interest at each time point.

The findings from this study suggest that freezing bovine colostrum at −20°C is a suitable method to preserve colostrum on-farm. However, given the observed reduction in IgG and insulin concentrations, colostrum should not be stored frozen for longer than 32 wk as a conservative recommendation. As our knowledge on other colostral components and the roles of each of these constituents in the neonatal calf expands, future investigations will be needed to refine the suitability of freezing colostrum as a storage method.
